# Oral health and quality of life: findings from the Survey of Health, Ageing and Retirement in Europe

**DOI:** 10.1186/s12903-022-02599-z

**Published:** 2022-12-15

**Authors:** Celina Block, Hans-Helmut König, André Hajek

**Affiliations:** Department of Health Economics and Health Services Research, University of Hamburg, Hamburg Center for Health Economics, Hamburg, Germany

**Keywords:** CASP, Missing teeth, Old age, Oral health, Quality of life, Successful ageing

## Abstract

**Background:**

The aim of this study was to clarify the link between oral health and quality of life among older adults in Europe.

**Methods:**

Cross-sectional data from wave 5 (n = 59,048 observations) were used from the representative Survey of Health, Ageing and Retirement in Europe. Oral health was quantified by three questions: presence of all natural teeth (yes; no); among individuals with missing natural teeth, the number of missing teeth and the extent of replaced natural teeth were quantified. Quality of life was quantified using the widely used CASP-12. Multiple linear regressions were used to determine the association between oral health and quality of life, adjusting for various potential confounders.

**Results:**

Multiple linear regressions showed that higher quality of life was associated with (1) the presence of all natural teeth and among individuals with missing natural teeth, with (2) a lower number of missing natural teeth and (3) completely replaced natural teeth. Additionally, quality of life was positively associated with younger age, being female, being married or in a partnership, higher income, higher educational level, not currently smoking, a lower number of functional impairments, better self-rated health, a lower number of depressive symptoms and a lower number of chronic diseases.

**Conclusion:**

Study findings showed an association between oral health and quality of life among older adults in Europe. Thus, the importance of good oral health for successful ageing was stressed. Future research is required to clarify the underlying mechanisms. Moreover, longitudinal studies are required to confirm our current findings.

## Background

Oral health refers to an unrestricted functionality as well as absence of inflammation and complaints of all organs of the oral cavity. This includes teeth, periodontium, mucous membranes, tongue, temporomandibular joints and salivary glands. As the oral cavity is the beginning of the digestive tract, oral health has a great significance for nutrition and a wide range of food products. Moreover, the masticatory organ is the place where the facial expressions arise which characterizes the individual’s identity and where the speech sound formation originates. A clear language as well as an aesthetic smile and aesthetic dentofacial profile may have an impact on social life like self-awareness and well-being of the own body [1–3].

In summary, there are many aspects of oral health to consider, but one particularly meaningful factor is the number of teeth which provide information about the functionality of the masticatory system, an aesthetic smile as well as an aesthetic profile (e.g., sunken face in toothless people).

Oral health represents a major concern for general health, while general health is a key determinant of overall quality of life. Oral health is a main part of general health since it has various effects on the entire organism [4, 5]. This can be explained by periodontitis contributing to the systemic inflammatory burden. There is a possible interaction between periodontitis with the complex pathogenesis of diabetes mellitus and cardiovascular disease as well as endocarditis and recurrent pneumonia in older age [6]. Good oral health may therefore contribute to the general health and the prevention of pathologies and may thus also affect overall quality of life [7].

Quality of life covers the subjective well-being in various areas of life. Drawing on the “Theory of Human Need” that acknowledges the social and biological components as equal, overall quality of life covers the dimensions Control, Autonomy, Self-realization and Pleasure (CASP). The “Theory of Human Need” is based on Maslow (1943), according to which individuals have an intrinsic motivation to fulfill a common set of needs.

*Control* is defined as the perception of being able to shape one’s own life, whereas *Autonomy* is described by self-determination. Moreover, *Self-realization* describes the fulfillment of oneself and finally *Pleasure* refers to the pursuit of enjoyable activities [8].

Demographically, we have focused on Europe as data of the World Health Organization showed that tooth decay among 6-year-old children in Europe varies from 20 to 90%. Moreover, in Europe among the general adult population over 50% suffer from some type of periodontal disease, while the prevalence of 60- to 65-year-olds varied from 70 to 85% in the year 2012 [9].

The fifth German Oral Health Study measured the average number of missing teeth in Germany in the year 2014 which among 65- to 74-year-olds was 11.1 teeth. Among 75- to 100-year-olds, individuals without care needs had an average number of missing teeth of 16.2 teeth and individuals in this age bracket with care needs had 22.8 missing teeth [10]. Moreover, 32.8% of the individuals aged 70 to 100 years were edentulous [10].

Oral health is of particular relevance among older adults, as the population continues to age [12]. Higher age is associated with more vulnerability and more physical and mental pathologies [13, 14] as well as more diseases and oral health problems [15]. These factors in turn may contribute to quality of life.

Apart from health, many determinants of quality of life among older adults have been examined in recent years including socioeconomic factors such as sex, age, education or income [16–18]. However, there is still very limited knowledge regarding the association between *oral health* and quality of life among older adults. For example, a systematic review has shown that a lower oral health is strongly associated with a lower quality of life among older institutionalized individuals [19]. Moreover, the number of lost teeth and the location of remaining teeth may have a great impact on quality of life [20].

Worth repeating: In light of the demographic ageing and because higher age is associated with more oral health problems [21], knowledge about this association is of high relevance. Due to the limited knowledge about the relationship between oral health and quality of life, our current study aimed to fill this gap based on representative data from various European countries.

The association between oral health and quality of life is also of great importance because exploring this relationship can assist in addressing individuals at risk for low quality of life. This knowledge may also stress the importance of good oral health for successful ageing as quality of life is positively associated with life satisfaction and successful ageing [22]. 

## Methods

### Sample

For the current study, cross-sectional data were used from the Survey of Health Ageing and Retirement in Europe (SHARE) study which started in 2004 (wave 1). For reasons of data availability, data were used from wave 5 in our study (which took place in the year 2013). Wave 5 is composed of first-time participants and individuals who already participated before.

The SHARE study is a widely known and large representative study. It includes community-dwelling individuals aged 50 and above (without an upper age limit) from 14 European countries (Austria, Belgium, Switzerland, Czech Republic, Germany, Denmark, Estonia, Spain, France, Italy, Luxembourg, Netherlands, Sweden, and Slovenia) and Israel in wave 5. Apart from these individuals, their potential partners (without age restrictions) were interviewed. The data collection is based on computer-assisted personal interviewing (CAPI) using a laptop or based on computer-assisted telephone interviewing (CATI).

In wave 5, the response rates were low (< 30%) only in Luxembourg, whereas moderate (30–40%) to high (50% and over) response rates were found in all other countries which were examined.

Further details regarding wave 5 are provided by Börsch-Supan et al. [23] and a general overview regarding the survey participation is provided by Bergmann et al. [24]. Moreover, additional details of the SHARE study in general have been given elsewhere [25].

During wave 1 to 4, SHARE was reviewed and approved by the Ethics Committee of the University of Mannheim, whereas wave 4 and the continuation of the project were reviewed and approved by the Ethics Council of the Max Planck Society. Furthermore, the country implementations of SHARE were reviewed and approved by the respective ethics committees or institutional review boards (if required).

### Dependent variable

We used the CASP-12 scale to quantify quality of life. It is a multidimensional measure of quality of life containing four domains (Control, Autonomy, Self-realization and Pleasure) [26]. CASP-12 is formed by 12 items which include the feelings and situations of the individuals who were interviewed (in each case: ranking from 1 = never to 4 = often) and which are summed to form the overall score. The range of the scale is thus from 12 which represents a very low quality of life across the four domains to 48 which represents a very high quality of life.

The CASP-12 is highly associated with the Life Satisfaction Index and the CASP-19 [27]. Furthermore, it has been shown that CASP-12 is a valid and reliable tool [28] for assessing the quality of life in old age and was also used in other large cohort studies [29].

### Key independent variable

In accordance with previous studies [19, 30–32], oral health can be measured by different dimensions, particularly the dental status is important for oral health.

The well-established dental status contains the number of teeth and the extent of replaced teeth which provide information about the functionality of the masticatory system as well as the aesthetical situation. The number of teeth reflects the extent of oral disease, since in most cases a pathological process such as periodontitis or caries accounts for tooth loss [33]. In addition, the number of teeth reflects the chewing ability which is an indicator of good oral health. However, this dimension does not represent pain which also characterizes a part of oral health. This should be acknowledged (please also see the limitations section).

Three questions were used to assess oral health. Oral health was quantified by (1) “Teeth are all natural.” (dichotomous: yes; no). The individuals without all natural teeth (i.e., answering the aforementioned question with “no”) were asked two more questions regarding oral health: (2) “How many teeth are missing?” (count score ranking from 1 to 30) and (3) the extent of replaced natural teeth (fully; partially; not at all).

### Covariates

Based on previous research [34, 35] and theoretical considerations (i.e. our thoughts on which factors might be relevant as covariates), covariates that can have an impact on quality of life were selected. A distinction was made between socioeconomic, lifestyle-related and health-related covariates – to cover main areas of life.

In regression analysis, socioeconomic covariates were selected as follows: age, sex (male; female), marital status (0 = married, living separated from spouse; never married; divorced; widowed; 1 = married and living together with spouse; registered partnership), household net income in Euro and educational level (0 = low educational level; 1 = medium educational level; 2 = high educational level) as well as the lifestyle-related covariate currently smoking (yes; no). Furthermore, it was adjusted for several health-related covariates, namely functional impairment (instrumental activities of daily living index, ranking from 0 to 3 with higher values corresponding to more difficulties, the scale was adapted from Lawton and Brody [28]), self-rated health (ranking from 1 = excellent to 5 = poor), depressive symptoms (Euro-D, ranking from 0 to 12 with higher values corresponding to more depressive symptoms) [36], chronic conditions (count score ranking from 0 to 10, including: heart attack; high blood pressure or hypertension; high blood cholesterol; stroke or cerebral vascular disease; diabetes or high blood sugar; chronic lung disease; cancer or malignant tumor; stomach or duodenal ulcer; peptic ulcer; Parkinson disease; cataracts; hip fracture or femoral fracture).

### Statistical analysis

Sample characteristics are first shown (stratified by the presence of all natural teeth). Moreover, effect sizes (Cohen’s d) for the association between the presence of all natural teeth and quality of life were computed. Additionally, multiple linear regressions were conducted to examine the association between oral health and quality of life (among all individuals and among individuals with at least one missing natural tooth). Statistical significance was defined as p value of 0.05 or smaller. Stata 16.1 (Stata Corp., College Station, Texas) was used to conduct statistical analyses.

## Results

### Sample characteristics

The sample characteristics (analytical sample, stratified by the presence of all natural teeth) are given in Table 1 (n = 59,048 individuals). In the total sample, mean age was 66.8 years (SD: 9.8 years, ranking from 50 to 112 years) with more than 55% being female. Furthermore, average quality of life score was 38.0 (SD: 6.2). In sum, 76.8% of the participants had at least one missing tooth, whereas 23.2% of the individuals reported having all natural teeth.

**Table 1:** Sample characteristics stratified by the presence of all natural teeth

Variables	Presence of all natural teeth	Absence of all natural teeth	Total
	N = 13,723	N = 45,335	N = 59,058
Quality of life (CASP-12): mean (SD)	39.6 (5.6)	37.5 (6.3)	38.0 (6.2)
Age in years: mean (SD)	62.8 (8.5)	68.0 (9.9)	66.8 (9.8)
Gender: n (%)			
Male	5791 (22.0%)	20,499 (78.0%)	26,290 (100.0%)
Female	7932 (24.2%)	24,836 (75.8%)	32,768 (100.0%)
Marital status: n (%)			
Married, living separated from spouse; never married; divorced; widowed	3290 (18.9%)	14,123 (81.1%)	17,413 (100.0%)
Married and living together with spouse; registered partnership	10,433 (25.1%)	31,212 (74.9%)	41,645 (100.0%)
Household net income (per year) in Euro: mean (SD)	41,500 (37.300)	32,200 (76.700)	34,400 (69.700)
Educational level: n (%)			
Low educational level	3850 (16.8%)	19,122 (83.2%)	22,972 (100.0%)
Medium educational level	5394 (24.2%)	16,911 (75.8%)	22,305 (100.0%)
High educational level	4479 (32.5%)	9302 (67.5%)	13,781 (100.0%)
Currently smoking: n (%)			
Yes	2038 (19.5%)	8402 (80.5%)	10,440 (100.0%)
No	11,685 (24.0%)	36,933 (76.0%)	48,618 (100.0%)
Functional impairment: mean (SD)	0.0 (0.2)	0.1 (0.4)	0.1 (0.3)
Self-rated health: mean (SD)	2.7 (1.1)	3.2 (1.1)	3.1 (1.1)
Depressive symptoms: mean (SD)	1.9 (2.0)	2.5 (2.3)	2.4 (2.2)
Chronic conditions: mean (SD)	0.8 (1.1)	1.3 (1.3)	1.2 (1.2)
Country			
Austria	657 (17.0%)	3219 (83.0%)	3876 (100.0%)
Germany	997 (18.8%)	4302 (81.2%)	5299 (100.0%)
Sweden	1994 (48.2%)	2141 (51.8%)	4135 (100.0%)
Netherlands	1088 (28.8%)	2692 (71.2%)	3780 (100.0%)
Spain	1092 (19.0%)	4669 (81.0%)	5761 (100.0%)
Italy	1085 (24.9%)	3281 (75.1%)	4366 (100.0%)
France	663 (16.1%)	3464 (83.9%)	4127 (100.0%)
Denmark	1643 (42.8%)	2201 (57.2%)	3844 (100.0%)
Switzerland	1143 (40.3%)	1693 (59.7%)	2836 (100.0%)
Belgium	860 (16.7%)	4277 (83.3%)	5137 (100.0%)
Israel	535 (31.8%)	1149 (68.2%)	1684 (100.0%)
Czech Republic	1039 (21.3%)	3830 (78.7%)	4869 (100.0%)
Luxembourg	301 (20.3%)	1183 (79.7%)	1484 (100.0%)
Slovenia	324 (11.9%)	2403 (88.1%)	2727 (100.0%)
Estonia	302 (5.9%)	4831 (94.1%)	5133 (100.0%)

Functional impairment: ranking from 0 to 3 with higher values corresponding to more difficulties

Self-rated health: from 1 = “excellent” to 5 = “poor”

Depressive symptoms: ranking from 0 to 12 with higher values corresponding to more depressive symptoms

Chronic conditions: count score from 0 to 10, with higher values reflecting more chronic conditions

Among individuals with at least one missing natural tooth, average number of missing teeth was 13.3 (SD: 10.0). Moreover, 51.0% of these individuals replaced some, 28.4% replaced all and 20.6% replaced none. Furthermore, Cohen’s d for the association between all natural teeth (dichotomous: yes or no) and quality of life (continuous) was 0.34.

### Regression analysis

Findings of multiple linear regressions are displayed in Table 2 (among all individuals) and Table 3 (among individuals with at least one missing natural tooth).

**Table 2:** Determinants of quality of life

Independent variables	Outcome measure: quality of life
Absence of at least one natural tooth (Refence category: Presence of all natural teeth)	− 0.22*** (0.05)
Age in years	− 0.00* (0.00)
Gender: Female (Reference category: Male)	0.52*** (0.04)
Marital status: married and living together with spouse; registered partnership (Reference category: Other^†^)	0.55*** (0.05)
Household net income (per year) in 1000 Euro	0.00* (0.00)
Education	
Medium educational level (Reference category: low educational level)	1.26*** (0.05)
High educational level	1.41*** (0.06)
Currently smoking (Reference category: yes)	0.13*** (0.01)
Functional impairment	− 1.20*** (0.07)
Self-rated health (from 1 = “excellent” to 5 = “poor”)	− 1.54*** (0.02)
Depressive symptoms	− 1.12*** (0.01)
Chronic conditions	− 0.08*** (0.02)
Constant	43.91*** (0.20)
Observations	59,058
R^2^	0.41

Findings of multiple linear regressions (with presence of all natural teeth as key independent variable; among individuals with at least one missing natural tooth)

Beta-coefficients (unstandardized) were reported; robust standard errors in parentheses; ***p < 0.001; **p < 0.01; *p < 0.05; + *p* < 0.10

^†^Other including: Married, living separated from spouse; never married; divorced; widowed

Functional impairment: from 0 to 3, higher values reflect more difficulties

Depressive symptoms: from 0 to 12, higher values reflect more depressive symptoms

Chronic conditions: count score from 0 to 10, with higher values reflecting more chronic conditions

**Table 3:** Determinants of quality of life

Independent variables	Outcome measure: quality of life
Number of missing natural teeth (count score ranking from 1 to 30)	− 0.03*** (0.00)
Extent of replaced natural teeth	
Partially (Reference category: Fully)	− 1.09*** (0.06)
Not at all	− 1.08*** (0.06)
Gender: Female (Reference category: Male)	0.45*** (0.05)
Marital status: married and living together with spouse; registered partnership (Reference category: Other^†^)	0.56*** (0.06)
Household net income (per year) in Euro	0.00* (0.00)
Education	
Medium educational level (Reference category: low educational level)	1.22*** (0.05)
High educational level	1.28*** (0.07)
Currently smoking (Reference category: yes)	0.11*** (0.02)
Functional impairment	− 1.08*** (0.07)
Self-rated health (from 1 = “excellent ” to 5 = ”poor”)	− 1.56*** (0.03)
Depressive symptoms	− 1.13*** (0.01)
Chronic conditions	− 0.08*** (0.02)
Constant	44.54*** (0.14)
Observations	44,578
R^2^	0.42

Findings of multiple linear regressions (with number of missing natural teeth and extent of replaced natural teeth as key independent variables; among individuals with at least one missing natural tooth)

Beta-coefficients (unstandardized) were reported; robust standard errors in parentheses; ***p < 0.001, **p < 0.01, *p < 0.05, + *p* < 0.10

^†^Other including: Married, living separated from spouse; never married; divorced; widowed

Functional impairment: from 0 to 3, higher values reflect more difficulties

Depressive symptoms: from 0 to 12, higher values reflect more depressive symptoms

Chronic conditions: count score from 0 to 10, with higher values reflecting more chronic conditions

Among all individuals displayed in Table 2, regressions revealed that lower quality of life was associated with the absence of at least one natural tooth (β = 0.22, *p* < 0.001).

Moreover, regression analysis among individuals with at least one missing natural tooth is displayed in Table 3. Regressions showed that lower quality of life was associated with a higher number of missing natural teeth (β = 0.03, *p* < 0.001). Additionally, higher quality of life was associated with fully extent of replaced natural teeth (compared to individuals who did not replace their missing natural teeth at all, β =  − 1.08, *p* < 0.001).

Regarding covariates (e.g. in Table 2), higher quality of life was associated with younger age, being female, being married or in a partnership, higher income, higher educational level, not currently smoking, lower functional impairment, a better self-rated health, a lower number of depressive symptoms and a lower number of chronic diseases.

In additional analysis, we also adjusted for country effects. Actually, there were some differences between the countries in terms of quality of life (results not shown, but available upon request). There was still an association between lower quality of life and the absence of at least one natural tooth (β =  − 0.04, *p* < 0.001). In another robustness check, we used log income (instead of income/1000). A higher log income was associated with higher quality of life (β = 0.30, *p* < 0.001). The association between lower quality of life and the absence of at least one natural tooth remained nearly the same.

## Discussion

Based on a large representative sample, the aim of this study was to clarify the association between oral health and quality of life among older adults in Europe.

Regressions showed that higher quality of life was associated with the presence of all natural teeth as well as a lower number of missing natural teeth and a fully extent of replaced natural teeth among individuals with at least one missing natural tooth. Our current study markedly extends the very limited knowledge regarding the association between oral health and quality of life among older adults.

In general, our current findings mainly confirm previous studies [19, 30, 31]. Previous studies based on other tools to quantify oral health showed that a low oral health can contribute to a lower quality of life [30], for example painful complaints [37, 38] or functional complaints like malocclusion could contribute to lower quality of life [39]. A systematic review also concluded that both painful or functional complaints are related to impaired quality of life [19].

To put in other words, our current findings are consistent with previous studies, however, we refer to the dental status by which we determine oral health. More precisely, we assume that oral health is measured by the number of natural teeth and the number of replaced teeth. First and foremost, functional and aesthetical complaints resulting from a lack of dental status are considered, whereas pain is not examined.

The association between lower oral health and lower quality of life which was found in our study may be explained as follows: Poor oral health causes a number of different limitations which in turn result in poor quality of life. These limitations can be functional, nutritional, aesthetical or psychological problems [31].

More precisely, first of all a low oral health measured by the dental status leads to a loss of function, this means a lower number of natural teeth or a lower number of replaced natural teeth commonly result in a poorer mastication. This is accompanied by more restrictions on food intake as well as more restrictions in nutrition in general. Malnutrition is associated with a lower quality of life [40].

Furthermore, large gaps between the teeth can be a problem in speech phonation. This can lead to restrictions in phonetics and thus also in communication. We assume that this in turn can contribute to a lower overall quality of life. In addition, tooth gaps in the front which are visible in the facial region may be an aesthetical limitation and thus could contribute to a low psychological well-being [3]. Moreover, a low oral health could be associated with low self-esteem [41]. Low self-esteem in turn could contribute to a low quality of life.

As previously mentioned, another outcome of a low oral health is the psychological dimension which can contribute to social isolation or loneliness [42]. This may be explained by various burdens which can have an impact on these factors, for example a masticatory problem, phonation or communication problem or an aesthetical limitation. It has been shown that a poor oral health could adversely affect the psychological state as well as social relationships [30, 31, 39]. This could contribute to low social participation, low social support and more loneliness which is a serious concern in aging populations [43]. This in turn could lead to a reduced quality of life [44].

Furthermore, it is necessary to point out the stigmatization of the society which accompanies a negative definition of the characteristic, in this case a high number of missing and not replaced teeth. Individuals with a low oral health may feel shame or a burden which is socially influenced and plays an essential role for a low quality of life [45, 46].

Our study refers to older adults from 50 years and above which is justified by a higher number of oral health problems. Oral diseases, particularly periodontal disease and dental caries are a widespread phenomenon with a higher prevalence in older age. With an increase in age, more dental plaque is found, paired with decreased poor general condition causing severe periodontitis [32]. For example, more dental plaque can cause gingival inflammation which in turn can contribute to severe periodontitis [47]. More precisely, more plaque caused by microorganisms leads to increased cytokine production which damages the dental epithelium. Subgingival plaque develops which is predominantly composed of gram-negative, anaerobic bacteria [48]. Impaired defense mechanisms cause the activation of osteoclasts which promote bone resorption.

Moreover, tooth loss and pain are outcomes of a severe periodontitis. The number of lost teeth and the location of remaining teeth may have a great impact on quality of life [20]. As our findings also indicate, a higher number of remaining teeth and fully prosthetic replacements are associated with a higher quality of life. Furthermore, it has been shown that the older population often pays more attention to their general health while sometimes ignoring the importance of oral health showing us the need to improve educational work [32]. In addition, it is necessary to consider that there are country specific differences in quality of life as well as in dental care. Future research is required to investigate this consideration.

Our results show that with partially replaced teeth, the quality of life is lower compared to full replacement. This means that a complete replacement of all missing teeth may particularly assist in maintaining quality of life in later life.

With regard to our covariates, our findings appear to be very plausible and were mostly in accordance with previous research [34, 49, 50]. For example, prior research has also shown that income as a socioeconomic covariate is associated with higher quality of life [49, 51]. Another example, it has been shown in prior research that smoking was associated with lower quality of life [50]. This can be explained by the various negative health-effects of smoking [50]. Moreover, it is well-known that health-related factors are associated with quality of life. For instance, it has been shown that functional impairment is associated with poorer quality of life [34, 52].

In this study, data were used from the large representative SHARE study which includes individuals from various European countries and Israel. Additionally, quality of life was quantified using the well-known and validated tool CASP-12 which was also used in other large cohort studies. To quantify oral health, three variables were used which provide a comprehensive insight referring to the number of natural teeth as well as the number of replaced teeth. This provides information about the functionality of the masticatory system as well as the aesthetical situation. However, painless and symptom free oral environment which correlates with a healthy tooth status was not measured in this study. Moreover, the individuals self-assessed these three questions about oral health which is not fully comparable to an objective assessment. The responses are characterized by personal experiences. Even though, the number of natural teeth is a reliable and objective tool. Further research with clinical data regarding oral health is therefore required to confirm our current findings. Additionally, a small sample selection bias has been identified in the SHARE study [36].

## Conclusion

Study findings showed an association between oral health and quality of life. This is of great importance for successful ageing among older adults in Europe. Efforts to maintain oral health should be encouraged in older age. Moreover, the importance of a complete dental status is of growing interest and every individual should have access to dental care to enable good oral health. In sum, tooth spaces should be provided by dental provision to maintain quality of life in later life.

Future research is required to clarify the underlying mechanisms. Additionally, potential moderators could be examined in future studies (e.g. sex, educational level or health literacy [53]).

## Data Availability

Data were obtained from a third party and cannot be made publicly available. More specifically, data for the study came from the SHARE project and are available to all researchers for purely scientific purposes upon request on their website (http://www.share-project.org/). Contact data: SHARE Research Data Center, c/o Centerdata, Tilburg University, P.O. Box 90,153, 5000 LE Tilburg, The Netherlands, Email: share-rdc@centerdata.nl.

## References

[1] Deng X, Wang YJ, Deng F, Liu PL, Wu Y (2018). Psychological well-being, dental esthetics, and psychosocial impacts in adolescent orthodontic patients: a prospective longitudinal study. Am J Orthod Dentofac Orthop.

[2] Shan B, Werger M, Huang W, Giddon DB (2021). Quantitating the art and science of esthetic clinical success. J World Fed Orthod.

[3] Venete A, Trillo-Lumbreras E, Prado-Gasco VJ, Bellot-Arcis C, Almerich-Silla JM, Montiel-Company JM (2017). Relationship between the psychosocial impact of dental aesthetics and perfectionism and self-esteem. J Clin Exp Dent.

[4] Johnson NW, Glick M, Mbuguye TN (2006). (A2) Oral health and general health. Adv Dent Res.

[5] Roleder J, Wilczyńska-Borawska M, Nowosielski C, Małyszko J (2016). Interdisciplinary nature of oral diseases–clinical implications. Przegl Lek.

[6] Dörfer C, Benz C, Aida J, Campard G (2017). The relationship of oral health with general health and NCDs: a brief review. Int Dent J.

[7] Fiorillo L. Oral health: the first step to well-being. Medicina (Kaunas). 2019;55(10). 10.3390/medicina55100676 PMC6843908 31591341

[8] Borrat-Besson C, Ryser V-A, Gonçalves J. An evaluation of the CASP-12 scale used in the Survey of Health, Ageing and Retirement in Europe (SHARE) to measure Quality of Life among people aged 50. Lausanne: FORS. 2015.

[9] Patel R. The state of oral health in Europe. Report commissioned by the platform for better oral health in Europe. 2012.

[10] Nitschke I, Hahnel S (2021). Dental care for older people: opportunities and challenges. Bundesgesundheitsblatt Gesundheitsforschung Gesundheitsschutz.

[11] Jordan AR, Micheelis W (2016). Fünfte Deutsche Mundgesundheitsstudie-(DMS IV).

[12] Harford J (2009). Population ageing and dental care. Commun Dent Oral Epidemiol.

[13] Mitchell E, Walker R (2020). Global ageing: successes, challenges and opportunities. Br J Hosp Med (Lond).

[14] Stewart TL, Chipperfield JG, Perry RP, Weiner B (2012). Attributing illness to 'old age:' consequences of a self-directed stereotype for health and mortality. Psychol Health.

[15] Islas-Granillo H, Borges-Yañez SA, Navarrete-Hernández JJ, Veras-Hernández MA, Casanova-Rosado JF, Minaya-Sánchez M (2019). Indicators of oral health in older adults with and without the presence of multimorbidity: a cross-sectional study. Clin Interv Aging.

[16] Eisele M, Kaduszkiewicz H, König HH, Lange C, Wiese B, Prokein J (2015). Determinants of health-related quality of life in older primary care patients: results of the longitudinal observational AgeCoDe Study. Br J Gen Pract.

[17] le Hoi V, Chuc NT, Lindholm L (2010). Health-related quality of life, and its determinants, among older people in rural Vietnam. BMC Public Health.

[18] Tajvar M, Arab M, Montazeri A (2008). Determinants of health-related quality of life in elderly in Tehran. Iran BMC Public Health.

[19] Wong FMF, Ng YTY, Leung WK (2019). Oral health and its associated factors among older institutionalized residents: a systematic review. Int J Environ Res Public Health.

[20] Tan H, Peres KG, Peres MA (2016). Retention of teeth and oral health-related quality of life. J Dent Res.

[21] Busby M, Chapple L, Matthews R, Burke FJ, Chapple I (2014). Continuing development of an oral health score for clinical audit. Br Dent J.

[22] Choi M, Lee M, Lee MJ, Jung D (2017). Physical activity, quality of life and successful ageing among community-dwelling older adults. Int Nurs Rev.

[23] Börsch-Supan AM, F. SHARE wave 5: innovations & methodology. Mannheim: MEA. 2015.

[24] Bergmann M, Kneip T, De Luca G, Scherpenzeel A. Survey participation in the survey of health, ageing and retirement in Europe (SHARE). Wave 1–7. Munich: SHARE-ERIC; 2019.

[25] Borsch-Supan A, Brandt M, Hunkler C, Kneip T, Korbmacher J, Malter F (2013). Data resource profile: the Survey of Health, Ageing and Retirement in Europe (SHARE). Int J Epidemiol.

[26] Blane D, Higgs P, Hyde M, Wiggins R (2004). Quality of life in the third age: key predictors of the CASP-19 measure. Ageing Soc.

[27] Blane D, Berney L, Smith GD, Gunnell DJ, Holland P (1999). Reconstructing the life course: health during early old age in a follow-up study based on the Boyd Orr cohort. Public Health.

[28] Kim GR, Netuveli G, Blane D, Peasey A, Malyutina S, Simonova G (2015). Psychometric properties and confirmatory factor analysis of the CASP-19, a measure of quality of life in early old age: the HAPIEE study. Aging Ment Health.

[29] Valdez R, Aarabi G, Spinler K, Walther C, Kofahl C, Buczak-Stec E (2021). Do postponed dental visits for financial reasons reduce quality of life? Evidence from the Survey of Health, Ageing and Retirement in Europe. Aging Clin Exp Res.

[30] van de Rijt LJM, Stoop CC, Weijenberg RAF, de Vries R, Feast AR, Sampson EL (2020). The influence of oral health factors on the quality of life in older people: a systematic review. Gerontologist.

[31] Spanemberg JC, Cardoso JA, Slob E, Lopez-Lopez J (2019). Quality of life related to oral health and its impact in adults. J Stomatol Oral Maxillofac Surg.

[32] Sheng X, Xiao X, Song X, Qiao L, Zhang X, Zhong H (2018). Correlation between oral health and quality of life among the elderly in Southwest China from 2013 to 2015. Medicine.

[33] Gerritsen AE, Allen PF, Witter DJ, Bronkhorst EM, Creugers NH (2010). Tooth loss and oral health-related quality of life: a systematic review and meta-analysis. Health Qual Life Outcomes.

[34] Conde-Sala JL, Portellano-Ortiz C, Calvó-Perxas L, Garre-Olmo J (2017). Quality of life in people aged 65+ in Europe: associated factors and models of social welfare—analysis of data from the SHARE project (Wave 5). Qual Life Res.

[35] Hamren K, Chungkham HS, Hyde M (2015). Religion, spirituality, social support and quality of life: measurement and predictors CASP-12(v2) amongst older Ethiopians living in Addis Ababa. Aging Ment Health.

[36] Börsch-Supan, Gruber AS. Release version: 7.1.0. SHARE-ERIC. 2020.

[37] Naito M, Yuasa H, Nomura Y, Nakayama T, Hamajima N, Hanada N (2006). Oral health status and health-related quality of life: a systematic review. J Oral Sci.

[38] Oghli I, List T, Su N, Häggman-Henrikson B (2020). The impact of oro-facial pain conditions on oral health-related quality of life: a systematic review. J Oral Rehabil.

[39] Sun L, Wong HM, McGrath CP (2017). Relationship between the severity of malocclusion and oral health related quality of life: a systematic review and meta-analysis. Oral Health Prev Dent.

[40] Norman K, Haß U, Pirlich M (2021). Malnutrition in older adults-recent advances and remaining challenges. Nutrients.

[41] Grecu AG, Balazsi R, Dudea D, Mesaros AS, Strimbu M, Dumitrascu DL (2019). Oral health related quality of life and self-esteem in a general population. Med Pharm Rep.

[42] Hajek A, König H-H (2021). The Association between Oral Health-Related Quality of Life, Loneliness, Perceived and Objective Social Isolation—Results of a Nationally Representative Survey. Int J Environ Res Public Health.

[43] Rouxel P, Heilmann A, Demakakos P, Aida J, Tsakos G, Watt RG (2017). Oral health-related quality of life and loneliness among older adults. Eur J Ageing.

[44] Ekwall AK, Sivberg B, Hallberg IR (2005). Loneliness as a predictor of quality of life among older caregivers. J Adv Nurs.

[45] Ernst J, Mehnert A, Dietz A, Hornemann B, Esser P (2017). Perceived stigmatization and its impact on quality of life-results from a large register-based study including breast, colon, prostate and lung cancer patients. BMC Cancer.

[46] Masnari O, Schiestl C, Rössler J, Gütlein SK, Neuhaus K, Weibel L (2013). Stigmatization predicts psychological adjustment and quality of life in children and adolescents with a facial difference. J Pediatric Psychol.

[47] Lang NP, Schätzle MA, Löe H (2009). Gingivitis as a risk factor in periodontal disease. J Clin Periodontol.

[48] Lovegrove JM (2004). Dental plaque revisited: bacteria associated with periodontal disease. J N Z Soc Periodontol.

[49] Kim H-J, Park S, Park S-H, Heo YW, Chang B-S, Lee C-K (2017). The significance of frailty in the relationship between socioeconomic status and health-related quality of life in the Korean community-dwelling elderly population: mediation analysis with bootstrapping. Qual Life Res.

[50] Goldenberg M, Danovitch I, IsHak WW (2014). Quality of life and smoking. Am J Addict.

[51] Huguet N, Kaplan MS, Feeny D (2008). Socioeconomic status and health-related quality of life among elderly people: results from the Joint Canada/United States Survey of Health. Soc Sci Med.

[52] Gift HC, Redford M (1992). Oral health and the quality of life. Clin Geriatr Med.

[53] Baskaradoss JK (2018). Relationship between oral health literacy and oral health status. BMC Oral Health.

